# Data on the removal of Optilan Blue dye from aqueous media using starch-coated green synthesized magnetite nanoparticles

**DOI:** 10.1016/j.dib.2019.104165

**Published:** 2019-06-21

**Authors:** Manuela Stan, Ildiko Lung, Maria-Loredana Soran, Ocsana Opris, Cristian Leostean, Adriana Popa, Florina Copaciu, Mihaela Diana Lazar, Irina Kacso, Teofil-Danut Silipas, Alin Sebastian Porav

**Affiliations:** a National Institute for Research and Development of Isotopic and Molecular Technologies, 67-103 Donat, 400293 Cluj-Napoca, Romania; b University of Agricultural Sciences and Veterinary Medicine, 3-5 Calea Mănăştur, Cluj-Napoca 400372, Romania

## Abstract

In this data article, we present supplementary data related to the research article entitled “Starch-coated green synthesized magnetite nanoparticles for removal of textile dye Optilan Blue from aqueous media” Stan et al., 2019. Data interpretations are included in the related research article Stan et al., 2019. The synthesized starch-coated Fe_3_O_4_ nanoparticles (ST-coated Fe_3_O_4_ NPs) were analyzed by scanning electron microscopy (SEM) and high resolution transmission electron microscopy (HRTEM) to illustrate the shape and surface coating of nanoparticles. Moreover, the Brunauer-Emmett-Teller (BET) technique was used to evidence starch deposition on magnetite nanoparticles. The obtained nanocomposites were used for adsorption of Optilan Blue (OB) in batch conditions and the optimum agitation speed and point of zero charge (pH_pzc_) were established. After OB adsorption on ST-coated Fe_3_O_4_ NPs, the nanocomposites were analyzed by transmission electron microscopy (TEM), X-ray diffraction (XRD) and Fourier-transform infrared spectroscopy (FTIR). The stability of starch coated Fe_3_O_4_ NPs in the acidic as well as alkaline pH was also evidenced by FTIR spectroscopy. In addition, to test the stability of ST-coated Fe_3_O_4_ NPs, leaching experiments were carried out. The experimental data were compared with isotherm and kinetic models in order to determine the most suitable for fitting.

**Table:** Specifications table

Subject area	*Environmental Engineering*
More specific subject area	*Adsorption*
Type of data	*Table, image, figure*
How data was acquired	*TEM/HRTEM/SEM (STEM Hitachi HD-2700), XRD (Bruker D8), VSM (Bruker ELEXSYS 500), FTIR (JASCO 6100), ICP-OES (Optima 5300DV, Perkin Elmer), Spectrophotometer (UV-VIS, T8)*
Data format	*Analyzed*
Experimental factors	*The effect of four parameters like initial concentration of OB, ST-coated Fe*_*3*_*O*_*4*_*NPs dosage, pH and temperature were examined. After adsorption, the adsorbent was separated with an external magnetic field and the residual dye was determined spectrophotometrically.*
Experimental features	*In the first step Fe*_*3*_*O*_*4*_*NPs were prepared and in the second step the ST-coated Fe*_*3*_*O*_*4*_*NPs were synthesized. The obtained nanocomposites used as adsorbents, were characterized by TEM, XRD, FTIR, BET and VSM techniques.*
Data source location	*Cluj-Napoca, National Institute for Research and Development of Isotopic and Molecular Technologies, Romania*
Data accessibility	*Data are included in this article*
Related research article	*Manuela Stan, Ildiko Lung, Maria-Loredana Soran, Ocsana Opris, Cristian Leostean, Adriana Popa, Florina Copaciu, Mihaela Diana Lazar, Irina Kacso, Teofil-Danut Silipas, Alin Sebastian Porav. Starch-coated green synthesized magnetite nanoparticles for removal of textile dye Optilan Blue from aqueous media. J Taiwan Inst Chem Eng 2019;100:65-73**[1]*.

**Table:** 

**Value of the data** • The starch-coated green synthesized magnetite nanoparticles exhibit a relatively good ability for dye adsorption, show good stability, and can be easily removed from aqueous solutions by magnetic separation. • The isotherms and kinetics fitting data will be useful for predicting and modeling the adsorption capacity and mechanism of OB removal by the ST-coated Fe_3_O_4_ NPs. • The data obtained show that ST-coated Fe_3_O_4_ NPs can be used as efficient adsorbents for removal of the OB textile dye from aqueous media.

## 1 Data

The presented data are supplementary the research article of *J Taiwan Inst Chem Eng* 2019; 100:65–73 [1].

**Fig. 1:**
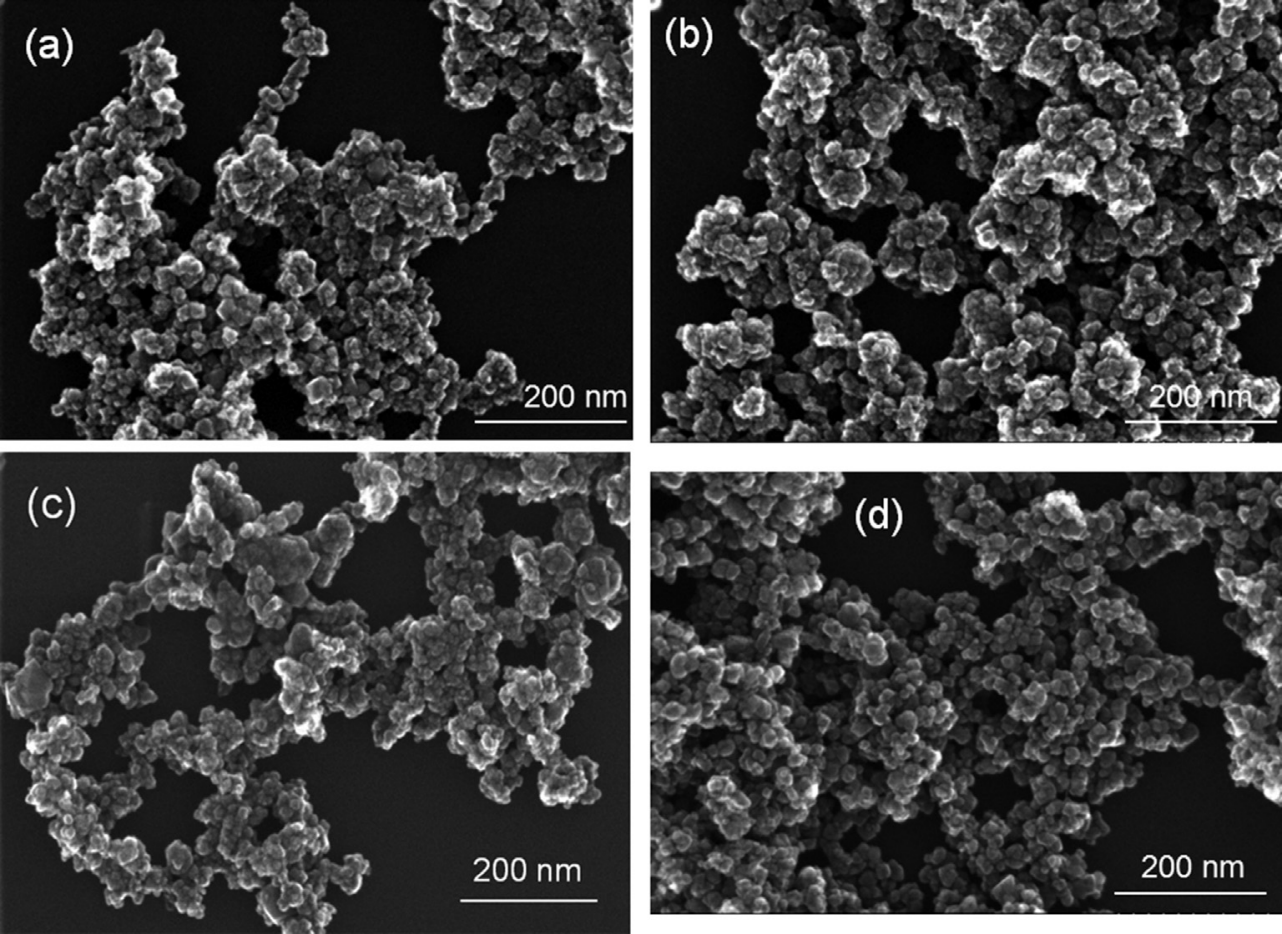
SEM images of ST-coated samples: (a) Fe_3_O_4_(av1), (b) Fe_3_O_4_(av2), (c) Fe_3_O_4_(wm) and (d) Fe_3_O_4_ samples.

**Fig. 2:**
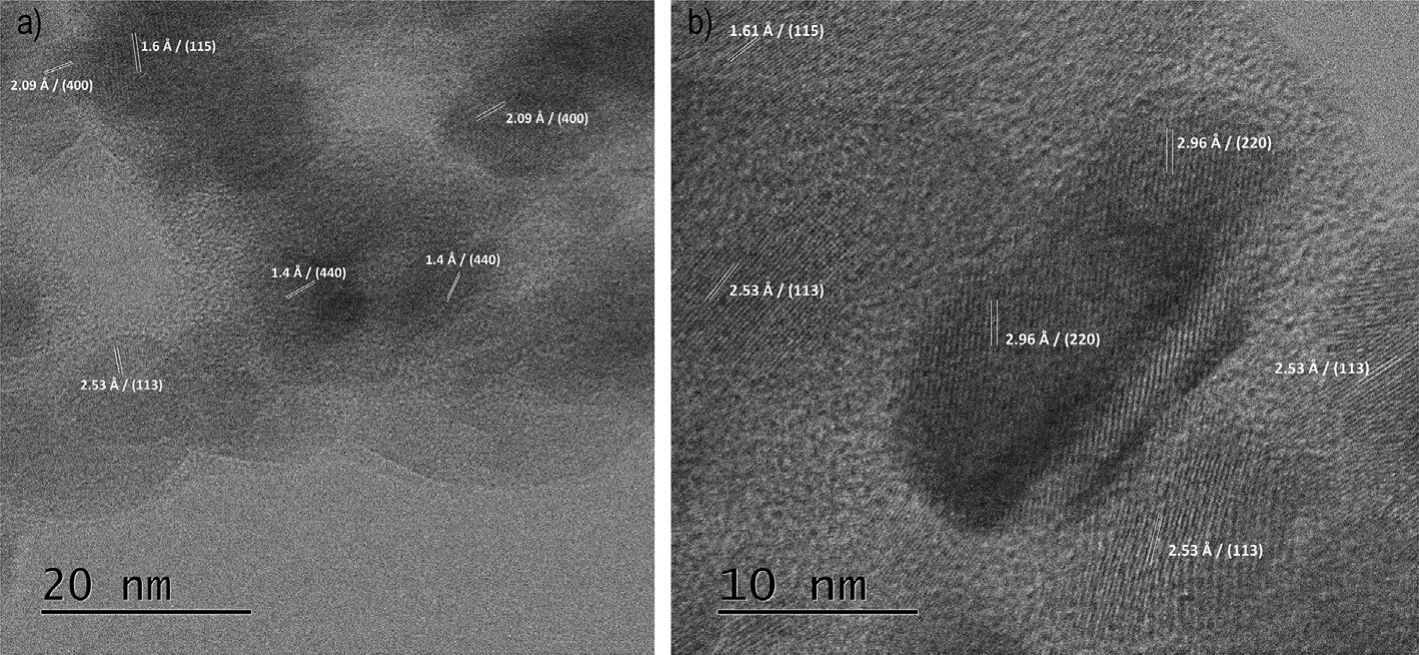
HR-TEM images of starch-coated: a) Fe_3_O_4_(av2) and b) Fe_3_O_4_ samples.

**Fig. 3:**
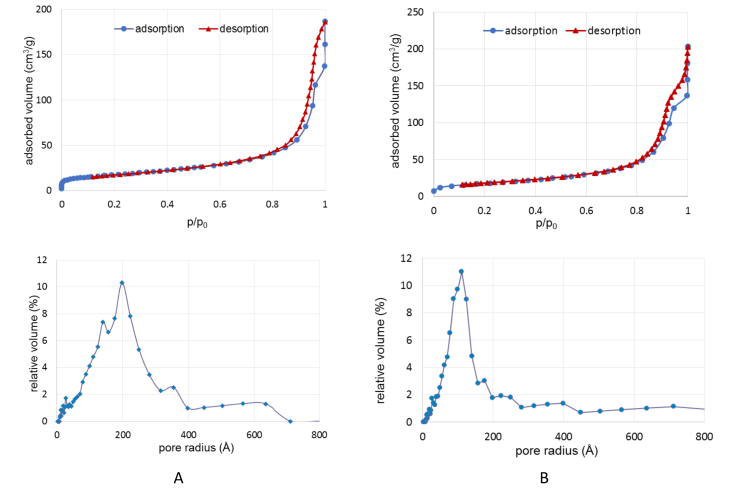
Nitrogen adsorption-desorption isotherms and pore radius for Fe_3_O_4_ sample. before (A) and after starch coating (B).

The XRD and TEM data for ST-coated Fe_3_O_4_ NPs after OB adsorption are summarized in Table 1. The adsorption capacity of the tested adsorbents was compared with other magnetic adsorbents used for dye removal in Table 2. Fig. 1 shows the SEM images of ST-coated Fe_3_O_4_ NPs, and the HRTEM images of two nanocomposite samples are illustrated in Fig. 2. The nitrogen adsorption-desorption isotherms and pore radius for Fe_3_O_4_ sample before and after starch coating are depicted in Fig. 3.

**Table 1:** The average size of crystallites (XRD) and nanoparticles (TEM) of starch-coated Fe_3_O_4_ NPs after OB adsorption.

Magnetite nanoparticles	Starch-coated magnetite nanoparticles after OB dye adsorption	
	XRD (nm)	TEM (nm)
Fe_3_O_4_(av1)^a^	14	19
Fe_3_O_4_(av2)	13	19
Fe_3_O_4_(wm)	14	16
Fe_3_O_4_	16	18

a Abbreviations used: av1 – avocado peel extract, av2 – avocado seed extract, wm – watermelon seed extract, no extract. Additional information can be found in the research article [1].

**Table 2:** Comparison of adsorption capacity/removal efficiency of various magnetic adsorbents for dyes.

Magnetic Adsorbent	Dye	Isotherm	Adsorption capacity (mg g^−1^)	Reference
Chitosan coated Fe_3_O_4_ nanoparticles	Reactive Yellow 145	Langmuir	47.62	[2]
Sodium alginate-coated Fe_3_O_4_ nanoparticles	Malachite green	Langmuir	47.84	[3]
PGA-coated Fe_3_O_4_ nanoparticles	Methylene blue	Langmuir	78.67	[4]
L-Serine functionalized Fe_3_O_4_ NPs	Rhodamine B	Langmuir	6.82	[5]
Magnetic Fe_3_O_4_@SiO_2_ starch-*graft*-poly (acrylic acid) (SPAA) nanocomposite hydrogels	Crystal violet	Langmuir	80.64	[6]
MNP@St-g-PVS	Methylene blue Malachite green	Langmuir	621 567	[7]
Lignin magnetic nanoparticles (LMNPs)	Methylene blue	Langmuir	211.42	[8]
Lignin amine magnetic nanoparticles (LAMNPs)	Acid scarlet GR	Langmuir	176.49	[8]
Fe_3_O_4_/Poly (styrene-co-methacrylic acid)	Crystal violet Rhodamine B	Langmuir	416.66 69.54	[9]
Chitosan coated Fe_3_O_4_ nanoparticles	Orange I	Langmuir	180.8	[10]
Starch-coated Fe_3_O_4_ NPs	Optilan blue	Langmuir	86–125	[1]

**Fig. 4:**
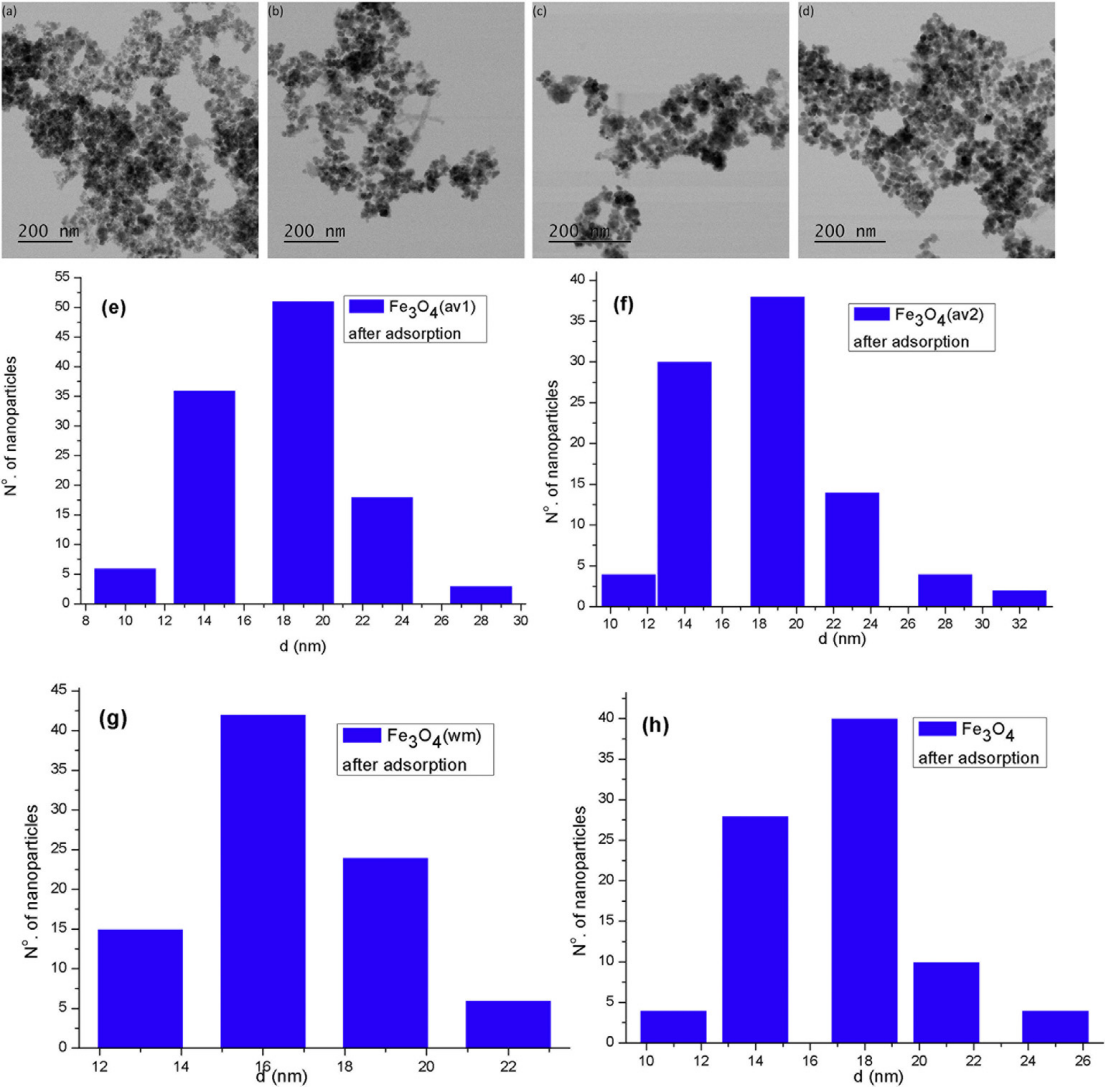
TEM images after OB adsorption of starch-coated: (a) Fe_3_O_4_(av1), (b) Fe_3_O_4_(av2), (c) Fe_3_O_4_(wm), (d) Fe_3_O_4_ samples and the histograms of particle size distribution (e–h).

**Fig. 5:**
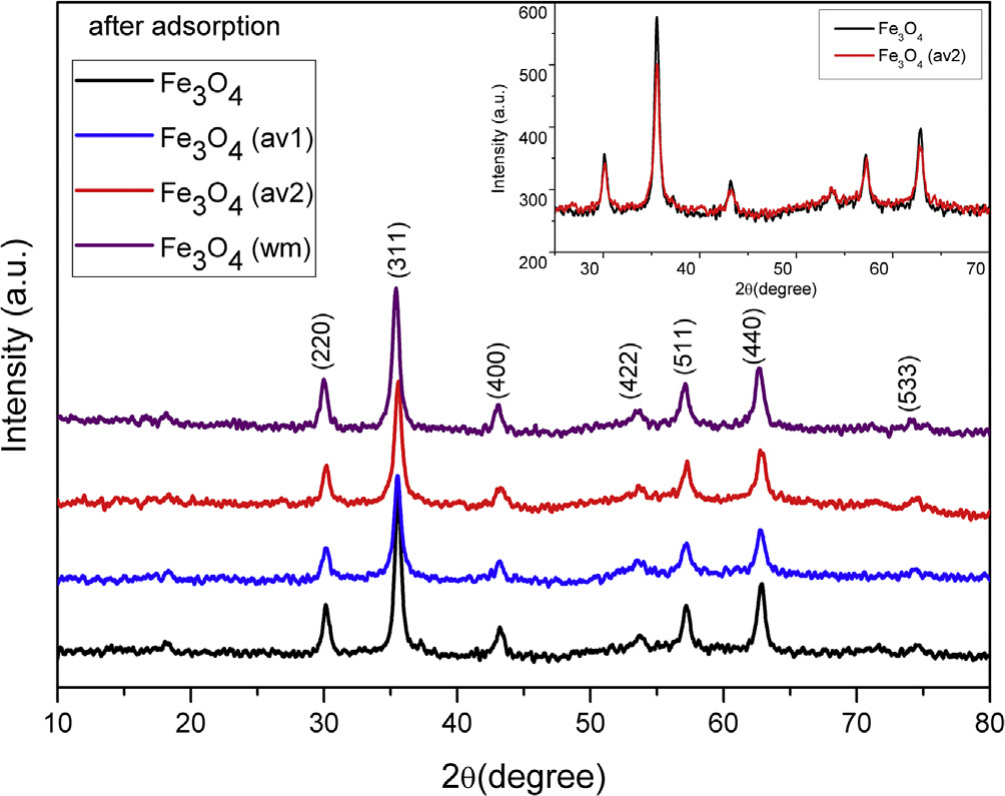
XRD patterns for ST-coated Fe_3_O_4_ NPs after adsorption of OB.

**Fig. 6:**
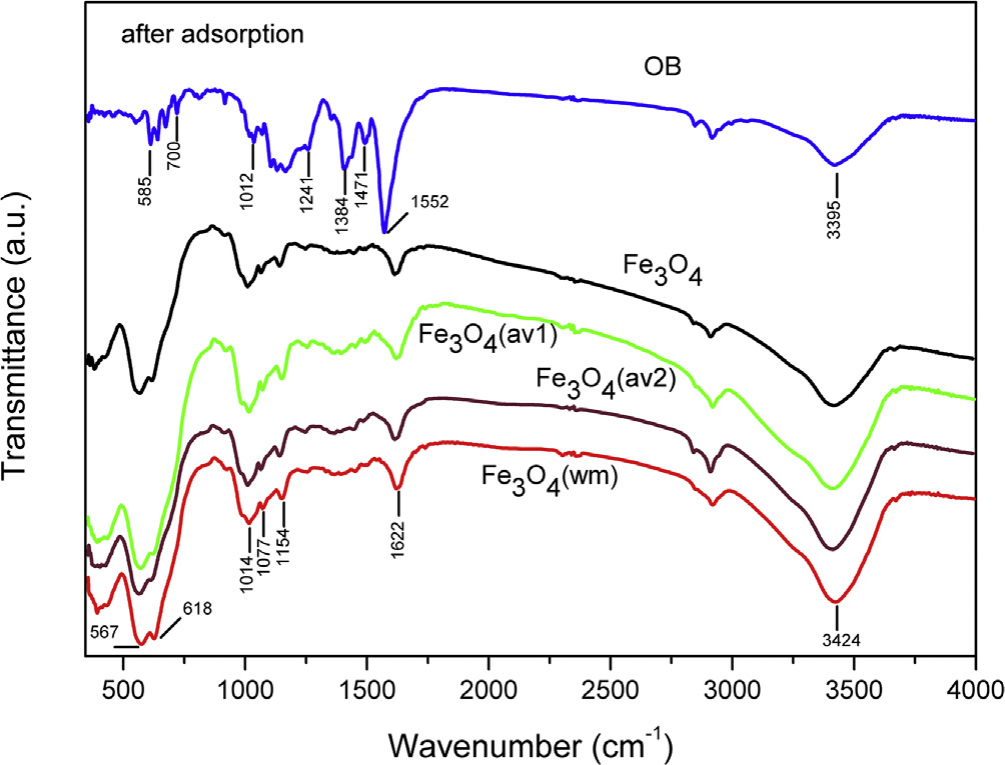
FTIR spectra of OB dye and ST-coated Fe_3_O_4_ NPs after dye adsorption.

**Fig. 7:**
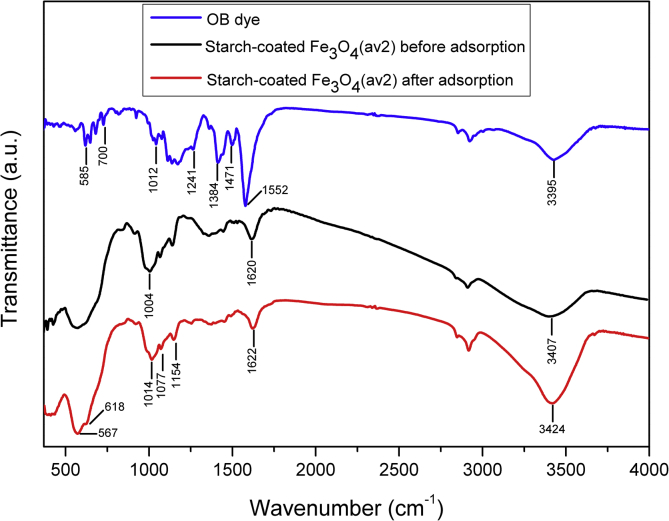
FTIR spectra of OB dye and starch-coated Fe_3_O_4_(av2) sample. before and after dye adsorption.

**Fig. 8:**
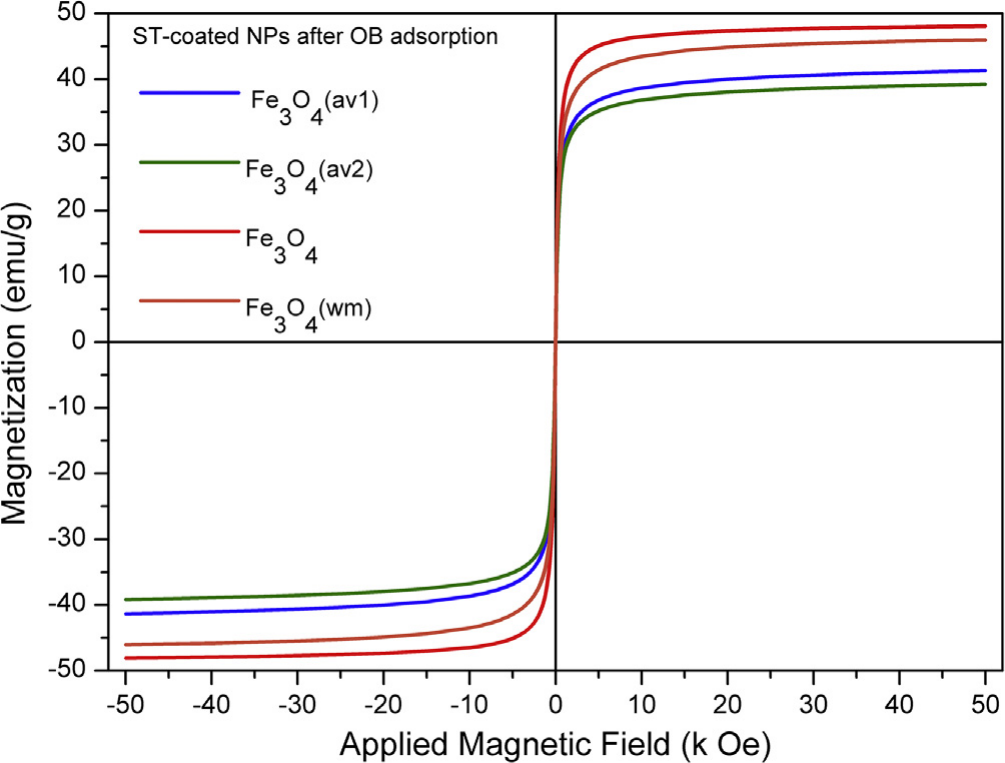
The behavior of magnetization vs. the applied magnetic field for ST-coated Fe_3_O_4_ NPs after OB dye adsorption.

**Fig. 9:**
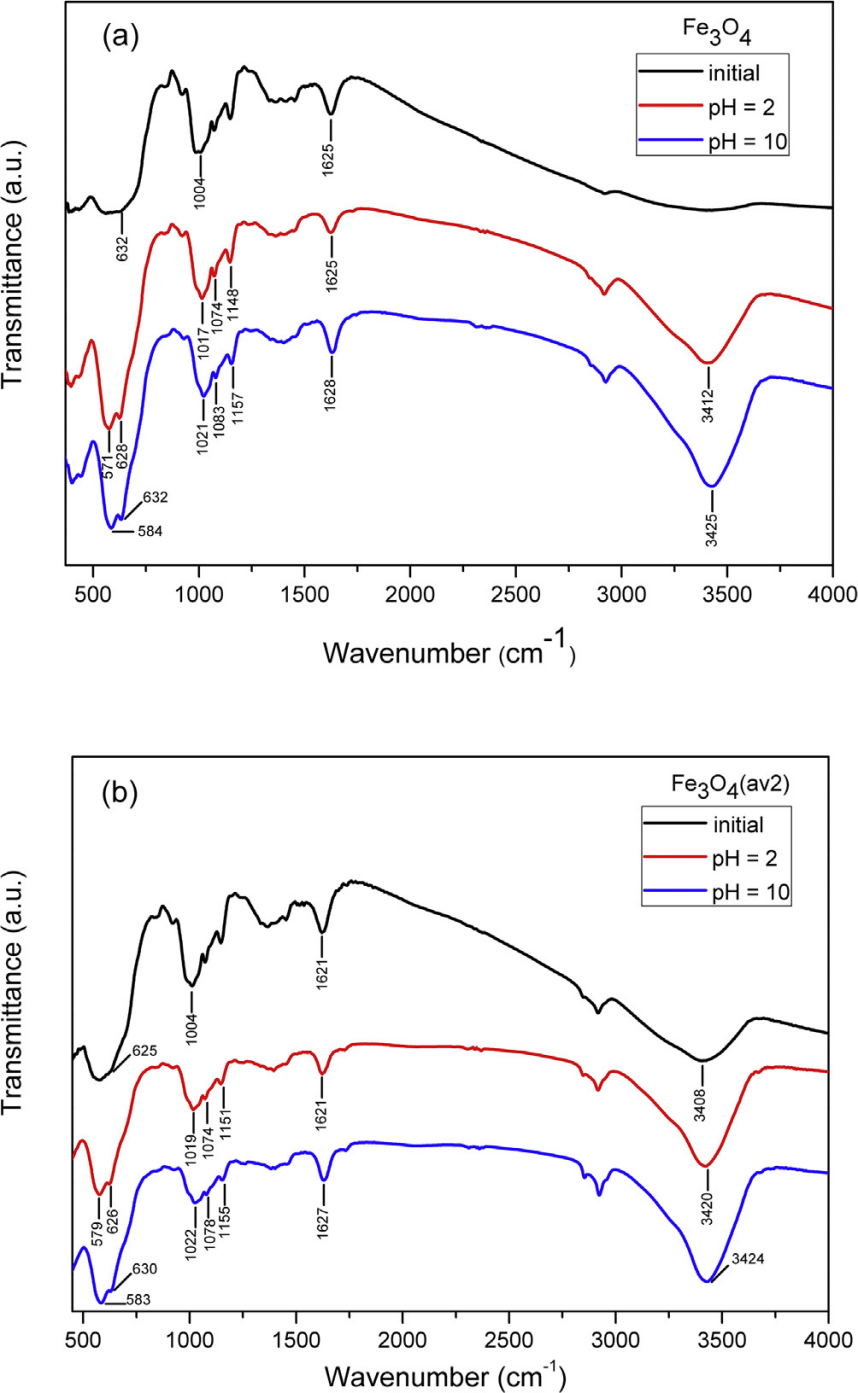
FTIR spectra of starch coated Fe_3_O_4_ and Fe_3_O_4_(av2) samples (a, b) in the acidic (pH 2) as well as alkaline (pH 10) media.

TEM, XRD, FTIR and VSM analyses of nanocomposites after OB adsorption are presented in Fig. 4, Fig. 5, Fig. 6, Fig. 7, Fig. 8. FTIR analysis was employed to demonstrate the stability of adsorbents in the acidic as well as alkaline media and the spectroscopic evidences are shown in Fig. 9.

**Fig. 10:**
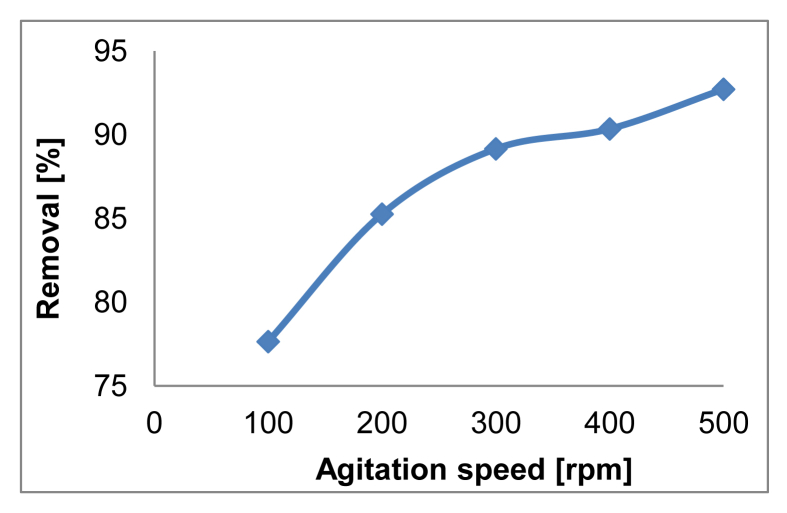
The effect of agitation speed on OB removal.

**Fig. 11:**
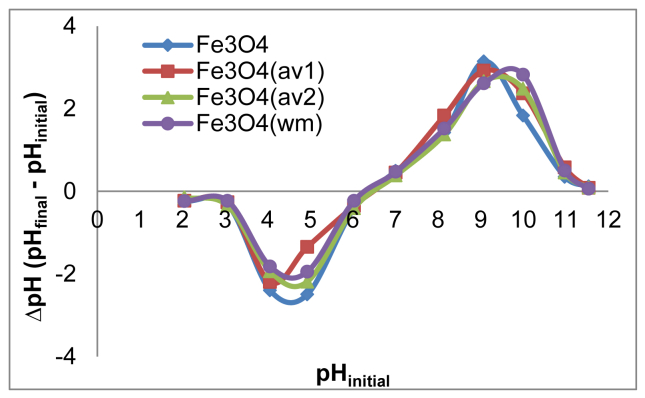
Point zero charge of ST-coated Fe_3_O_4_ NPs.

**Fig. 12:**
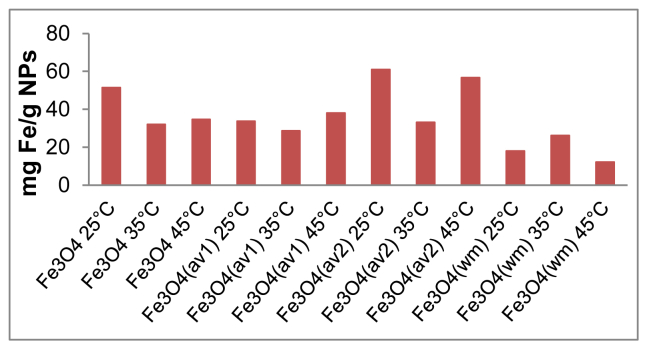
The total dissolved iron concentrations.

**Fig. 13:**
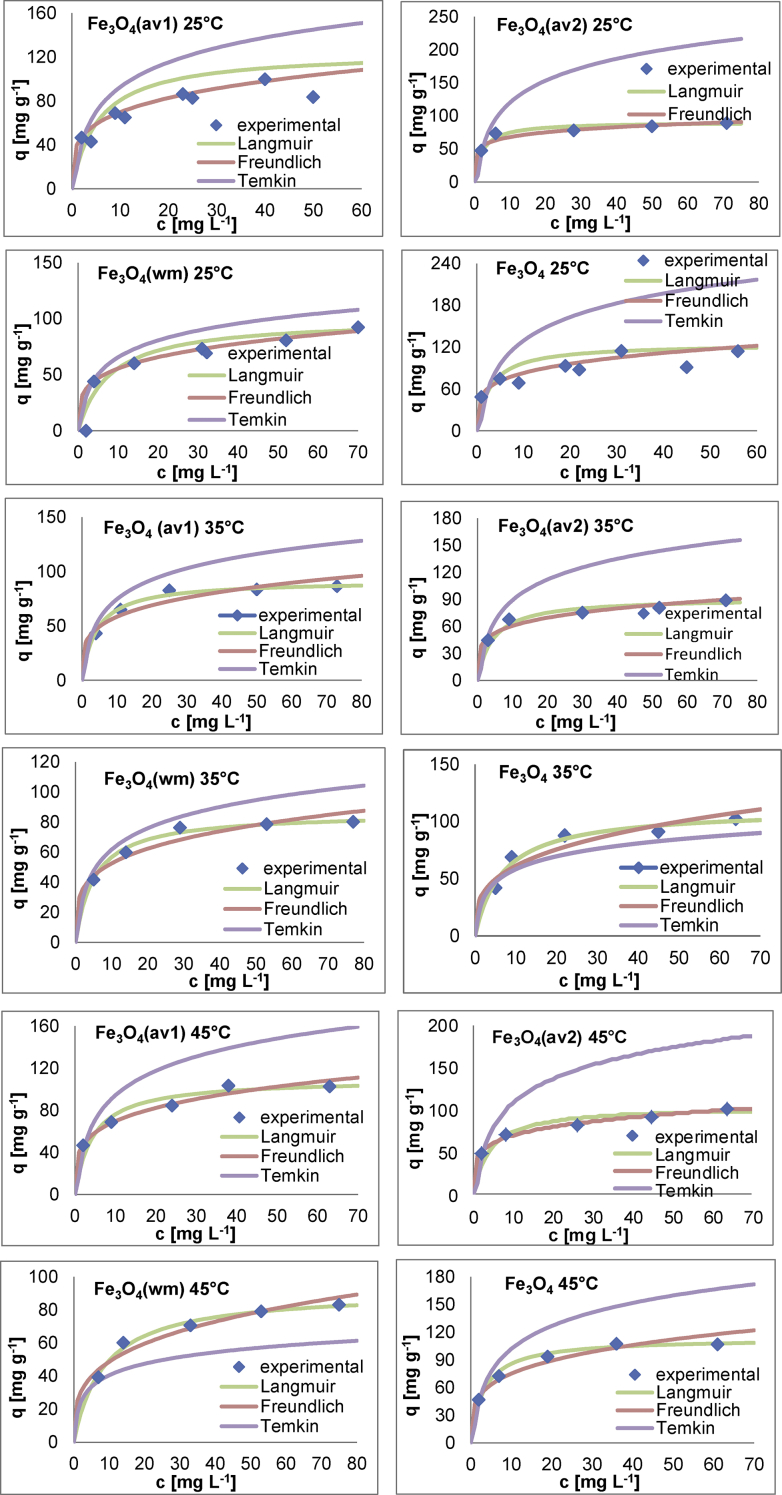
Adsorption isotherm models fitted to experimental adsorption of OB on ST-coated Fe_3_O_4_ NPs at different temperatures (pH 2, mass dosage 0.6 g L^−1^).

**Fig. 14:**
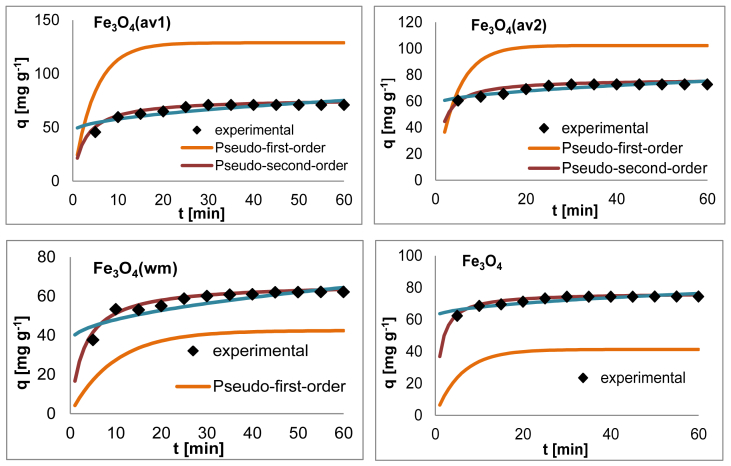
Adsorption rate curves (pH 2, dye conc. 50 mg L^−1^, 308 K and adsorbent dose 0.6 g L^−1^).

The effect of agitation speed on OB removal is presented in Fig. 10. The pH_pzc_ values for all adsorbents are determined by the position where the resulting curves cut through the pH_initial_ axis as observed in Fig. 11. Data from leaching experiments, carried out in order to establish complete magnetic separation and the total dissolved iron concentrations by ICP-OES analysis, are shown in Fig. 12. As illustrated in Fig. 13 and Fig. 14, adsorption isotherms and kinetics were modeled and fitted with experimental data in order to determine the interactions that occur between the adsorbent and adsorbate species, the adsorption rate by the adsorbent, and the adsorption mechanism of the solute onto an adsorbent.

## 2 Experimental design, materials, and methods

### 2.1 Materials

Starch-coated Fe_3_O_4_ NPs, Optilan Blue MF-GL dye, 0.5 N HCl or 5% NH_4_OH for pH adjustments.

### 2.2 Determination of pH_pzc_ of ST-coated Fe_3_O_4_ NPs

The pH drift method [11] was used to determine the pH at point of zero charge (pH_zpc_) of the adsorbents under study. Over 12 mg of adsorbent, 20 mL of 0.01 M NaCl solution was added with the initially pH adjusted in the range of 2–12 by adding 0.5 N HCl or 5% NH_4_OH. The mixtures were left at room temperature for 48 h, after which the solid material was separated from the solution using an external magnet and the final pH value was measured.

### 2.3 Adsorption experiments

Optilan Blue adsorption on ST-coated Fe_3_O_4_ NPs was performed under batch conditions. The effect of initial dye concentration, pH, temperature and adsorbent dosage on adsorption of OB on ST-coated Fe_3_O_4_ NPs were determined. In addition, a study was conducted to determine the optimum agitation speed (100–500 rpm) at which the maximum dye adsorption was accomplished. The solution was adjusted with 0.5 N HCl or 5% NH_4_OH in order to achieve the desired pH. The adsorbent was separated using an external magnet and the residual dye was measured with a UV–Vis spectrophotometer, recording the absorbance at 629 nm. The obtained experimental data were fitted to different models in order to understand the adsorption behavior of OB on ST-coated Fe_3_O_4_ NPs.

In addition, the stability of adsorbents in acidic (pH 2) and alkaline (pH 10) media was investigated.

### 2.4 Characterization and analysis

The surface morphology of nanocomposites was examined from SEM and HRTEM images. BET measurements were also conducted, and evidenced the deposition of starch on the surfaces of magnetite nanoparticles.

The stability of adsorbents was investigated in the acidic pH (2) as well as alkaline pH (10) by FTIR technique in order to underline the possible spectroscopic evidences.

After OB adsorption on ST-coated Fe_3_O_4_ NPs, the dried samples were characterized by different techniques such as FTIR, XRD, TEM, and VSM.

### 2.5 Batch leaching test

For each leaching test, 10 mg of ST-coated Fe_3_O_4_ NPs were mixed with 10 mL of water at pH 2 (HCl 0.1 M) in a 20 mL conical flask. The mixture was stirred with 500 rpm for 2 hours at different temperature (25 °C, 35 °C and 45 °C). The nanoparticles were separated using an external magnet and the leachate samples were analyzed by a dual viewing inductively coupled plasma optical emission spectrometer (ICP-OES). The experiment was performed in triplicate.
